# The Old and the New on Viral Diseases in Sturgeon

**DOI:** 10.3390/pathogens9020146

**Published:** 2020-02-21

**Authors:** Davide Mugetti, Paolo Pastorino, Vasco Menconi, Claudio Pedron, Marino Prearo

**Affiliations:** 1 Istituto Zooprofilattico Sperimentale del Piemonte, Liguria e Valle d’Aosta, 10154 Torino, Italy; paolo.pastorino@izsto.it (P.P.); vasco.menconi@izsto.it (V.M.); marino.prearo@izsto.it (M.P.); 2 Dipartimento di Scienze della Vita, Università degli Studi di Trieste, 34127 Trieste, Italy; 3 Independent researcher, 20090 Settala, Italy; claudio.pedron@alice.it

**Keywords:** sturgeon nucleocytoplasmic large DNA viruses, herpesviruses, white sturgeon adenovirus, *Acipenser* spp., sturgeon farm

## Abstract

Although sturgeon production by aquaculture has increased worldwide, a major factor limiting its expansion are infectious diseases, although few data about viral diseases are available however. This review provides a rapid overview of viral agents detected and described to date. Following a general introduction on viral diseases are four sections arranged by virus classification: sturgeon nucleocytoplasmic large DNA viruses, herpesviruses, white sturgeon adenovirus 1, and other viruses. Molecular diagnosis is currently the best tool to detect viral diseases, since cell culture isolation is not yet applicable for the detection of most sturgeon viruses.

## 1. Introduction

Sturgeon are known to be more resistant than other species to certain diseases in nature since they are anadromous species [1]. The economic importance of the *Acipenseridae* family is linked chiefly to caviar production [2]. Over the years, sturgeon have been decimated by overfishing and loss of their natural habitat [3,4]. The introduction of allochthonous species and widespread river pollution have further worsened their survival in the wild [5]. Breeding sturgeon in intensive facilities has increased meat and caviar production [6,7,8], but also raised problems with farming methods. One of the factors limiting the expansion of sturgeon farming are infectious diseases. While bacterial and parasitic diseases affect sturgeon [9,10], virosis seems to be the worst problem [1].

Intensive sturgeon farming is a relatively recent development, as is the study of viral diseases in sturgeon. Several viral agents are known to infect sturgeon (Table 1) and cases of non-specific sturgeon viruses in *Acipenseridae* have also been reported. Nonetheless, large gaps in viral biology and epidemiology remain. This review presents an overview of viral agents found in sturgeon and described in the literature. The life cycle of viruses is beyond the scope of this review. The aim is to provide a concise summary of the current knowledge on viral diseases and diagnostic analytical techniques.

## 2. Sturgeon Nucleocytoplasmic Large DNA Viruses (sNCLDV)

Sturgeon Nucleocytoplasmic Large DNA Viruses (sNCLDV) represent the most numerous and heterogeneous group of viral agents causing disease in sturgeon. The current nomenclature can lead to error in taxonomy [42]. Most viruses are identified as “iridovirus”, which may suggest that they belong to the *Iridoviridae* family; however, only frog virus 3 (FV3) is officially recognized as a member of *Iridoviridae* [43]. The other sNCLDV, not yet officially recognized by the International Committee on Taxonomy of Viruses (ICTV), have shown closer similarity with *Mimiviridae* [44]. This family includes a variety of large, double-stranded DNA viruses affecting eukaryotic organisms, mainly algae and protists. Should sNCLDV eventually be confirmed *Mimiviridae*, they will be first viruses in this family capable of infecting vertebrate organisms [45].

### 2.1. White Sturgeon Iridovirus (WSIV)

White sturgeon iridovirus (WSIV) is currently the sNCLDV for which more information is available; it poses a considerable problem for the conservation of endangered *Acipenseridae* species and for intensive aquaculture [42]. The ICTV has not yet assigned an official name to the virus. The name derives from the first report, in which mortality cases involving white sturgeon (*Acipenser transmontanus*) juveniles from several northern California farms were reported [11]. Diagnosis of WSIV was made by electron microscopy. The same research team isolated the virus on a cell monolayer (WSS-2). The other cell lines (WSH-1, CHSE-214, EPC) that were tested produced negative results. Although WSS-2 cells allowed visualization of WSIV, limitations were the onset of cytopathic effects and the slow virus growth [15]. Other mortality outbreaks in North America followed, making the virus a major concern for sturgeon farming [12,13,19]. WSIV was detected in juveniles and larger fish (up to about 50 cm). Studies have helped to clarify the importance of WSIV for the expansion of intensive sturgeon farming; briefly:

- The different sizes of sturgeon positive for the virus suggest a possible vertical transmission and adults as WSIV carriers [14,46,47];
- Not only white sturgeon, but other species belonging to the *Acipenseridae* family can also be susceptible (experimentally infected *A. fulvescens*) [15];
- Intensive breeding conditions (e.g., high density) are predisposing factors for disease onset [47];
- WSIV is considered an endemism of several American basins (Sacramento-San Joaquin, Columbia, Snake, Kootenai and Fraser rivers), which also poses a problem for stocking programs [12,14,19].

With regard to the host spectrum, a recent work highlighted WSIV DNA in various sturgeon species (*A. ruthenus*, *A. baerii*, *A. gueldenstaedtii*, *A. oxyrinchus*, *A. sturio*) and hybrids sampled between 2010 and 2014. In addition, positive cases were reported for Europe, signaling an expansion of the distribution area of WSIV [16]. There have been no reports of WSIV infection in other Teleost species [42].

External clinical signs include lethargy and lack of feeding [11,12], the latter of which is the primary cause of death and results from destruction of sensory epithelial tissue by the virus [48]. Mortality rates are higher in small sturgeon and are related to adverse breeding conditions (stress, temperature changes, overcrowding, manipulations); mortality due to WSIV can occur in adults also, although more contained [49]. Correct sampling includes the gills, mouth and fleshy portions of the fins to detect lesions or the virus [42]. WSIV diagnosis can be performed using molecular methods, either conventional PCR [50] or TaqMan Real-time PCR [16]. Histology, electron microscopy and cell culture isolation are also used [15]. In-situ hybridization, because of its low sensitivity, is not recommended for diagnostic purposes [16].

### 2.2. Missouri River Sturgeon Iridovirus (MRSIV)

Missouri River sturgeon iridovirus (MRSIV) is closely related to WSIV and is uniquely associated with two endemic *Acipenseridae* species of the Missouri and the Mississippi River: the pallid sturgeon (*Scaphirhynchus albus*) and the shovelnose sturgeon (*S. platorynchus*) [17]. The viruses identified in the two sturgeon species were initially named differently: shovelnose sturgeon iridovirus (SSIV) and pallid sturgeon iridovirus (PSIV). This differentiation was eventually abandoned and the two species are now considered variants of MRSIV [51]. Although it does not appear to create a problem for intensive sturgeon farms, MRSIV can pose a limit for restoration programs of pallid sturgeon and shovelnose sturgeon, indicated as endangered [52] and vulnerable [53] species, respectively, on the International Union for Conservation of Nature (IUCN) Red List.

Outbreaks in hatchery-reared juveniles of both species occurred in 1999. Symptoms included lethargy, emaciation and skin and fins inflammation; the mortality rate of several shovelnose sturgeon batches was 100%. Although virions have been detected by electron microscopy in epithelial cells, MRSIV has not been isolated on cell monolayer [17]. Rapid virus diagnosis and quantitation can be done using either conventional PCR or TaqMan Real-time PCR [51].

### 2.3. Shortnose Sturgeon Virus (SNSV)

Shortnose Sturgeon Virus (SNSV) is another pathogen affecting shortnose sturgeon (*A. brevirostrum*). The only published report in the literature is by La Patra and co-authors [18] and describes a case of co-infection with herpesvirus and irido-like virus in adult shortnose sturgeon from Atlantic coast Canada. Diagnosis was based on histology and electron microscopy. In a later phylogenetic study using PCR assays on infected formalin-fixed gills, the virus was named SNSV [54].

### 2.4. British Columbia White Sturgeon Virus (BCWSV)

The one report concerning British Columbia white sturgeon virus (BCWSV) dates from 2001 in white sturgeon (*A. transmontanus*) bred in British Columbia, Canada. The sturgeon (8–10 cm length) presented chronic inappetence, lethargy and lack of reactivity; mortality was reported, although the rate was not specified. Diagnosis was based on electron microscopy [19]. Available BCWSV gene sequences derive from the phylogenetic study by Clouthier and collaborators [54].

### 2.5. Namao Virus (NV)

Namao virus (NV) has been recognized as the causative agent of a mortality outbreak involving hatchery reared juvenile’s lake sturgeon (*A. fulvescens*) that occurred in Manitoba, Canada, in 2009 and 2010 [20]. The symptoms were inappetence, anorexia, erratic behavior, skin redness and bleeding and excess gill mucus. NV was identified by PCR amplification of a 219 bp portion of the major capsid protein gene. Moreover, cytopathic effects were detected in cell cultures from Manitoba lake sturgeon, but no changes were seen following the inoculation of NV on other cell lines (WSSK-1, WSS-2, CHSE-214, EPC). Electron microscopy revealed hexagonal virions in epithelial cells [20].

The only report of NV infection is by Clouthier and co-authors [20]. Other studies on NV focus mainly on the hypothesis that this and other sNCLDV belong to the *Mimiviridae* family [44,45,54].

### 2.6. Acipenser Iridovirus-European (AcIV-E)

Research into sNCLDV has increased only recently in Europe. Pending official nomenclature, the causative agent of mortality events in European sturgeon has been named acipenser iridovirus-European (AcIV-E) [21]. An initial report of an irido-like virus in Russian sturgeon (*A. gueldenstaedtii*) in Northern Europe dates from the 1990s [55]; the four-month-old fish were reared on a farm with a history of mortality events. Nonetheless, no in-depth investigation was carried out to identify the etiological agent. New mortality outbreaks in several European countries have involved four species of farm-bred sturgeon (*A. gueldenstaedtii*, *A. baerii*, *A. naccarii*, *Huso huso*) [21,22]. Ciulli and co-authors named the virus European sturgeon NCLDV [22], while other authors named it AcIV-E [21]; despite the difference, they appear to be several strains of the same virus [44]. Although the causative agent is the same, clinical manifestations differ in episodes and species of *Acipenseridae*. Moreover, discrepancies can also be found in a single species (e.g., Russian sturgeon), in size and symptoms: anorexia, lethargy and erratic swimming. Externally, skin ulcers and gills with abundant mucus can be noted, but no specific clinical signs. The virosis appears to affect juveniles more severely and the mortality rate is extremely variable (from 30% to 100%). Moreover, co-infection with opportunistic pathogens or environmental bacteria (*Aeromonas* spp., *Acinetobacter* spp., *Flavobacterium* sp., *Enterobacter* sp., *Chryseobacterium indologenes*, *Serratia liquefaciens*, *Hafnia alvei*) can occur [21,22]. Despite the many unknowns, Russian sturgeon seems to be can be the species most susceptible to AcIV-E infection. There is some evidence supporting this hypothesis [23,56,57]. New cases have been reported for other countries (Sweden) [24] and other species (*A. stellatus*, *A. ruthenus*) [25].

Unlike most cases described by American authors, AcIV-E was diagnosed primarily with PCR assays. Histological analysis was also performed, while it was not possible to isolate the virus on cell monolayers using several cell lines (BF-2, CCB, EPC, GF, SSN-1, E11, WSSK) [21,22].

### 2.7. Frog Virus 3 (FV3)

Several sNCLDV are named “Iridovirus”, although they are actually more similar to *Mimiviridae*. The exception is Frog Virus 3 (FV3), the only member of the *Iridoviridae* family currently known to cause infection in sturgeon. FV3 is officially recognized by the ICTV as the type species of the genus *Ranavirus* (family *Iridoviridae*, subfamily *Alphairidovirinae*) [43]. Sturgeon are not the only fish infected by FV3. Mortality in threespine stickleback (*Gasterosteus aculeatus*) has also been reported [58]. The case regarding the *Acipenseridae* family referred to a mortality outbreak in pallid sturgeon juveniles (*S. albus*) reared in Missouri (USA). In this epizootic event the cumulative mortality was 95%. The causative agent was identified as FV3 based on histology, electron microscopy and biomolecular assays [26]. No other reports followed this first case in sturgeon.

## 3. Herpesviruses

Herpesviruses, specifically the species belonging to the *Alloherpesviridae* family, are known fish pathogens [59,60,61]. There are also herpesviruses capable of infecting sturgeon, in America and in Europe. This has made herpesvirosis, like sNCLDV infection, the main viral disease in both farmed and wild sturgeon. Several species of these double-stranded DNA viruses that can specifically infect *Acipenseridae* have been described. Only one of them is officially recognized by the ICTV: acipenserid herpesvirus 2 (AciHV-2), which is included in the genus *Ictalurivirus* [31]. Non-sturgeon-specific *Alloherpesviridae* have also been reported [34,35].

### 3.1. Acipenserid Herpesvirus 1 (AciHV-1)

Acipenserid herpesvirus 1 (AciHV-1) was the first herpesvirus to be identified in sturgeon. The first case was reported in 1989 in California-bred white sturgeon (*A. transmontanus*) juveniles [27]. AciHV-1 produced syncytia and was isolated from a specific epithelial sturgeon cell monolayer (WSSK-1). Virus isolation on other cell lines (WSH-1, WSS-2, CHSE-214, EPC) yielded negative results. Diagnosis was based on by histology and electron microscopy; conspicuous features were necrotic lesions on skin epithelial tissue and virions with hexagonal capsid referable to herpesviruses. Nonetheless, no external clinical signs were evident until death. Other AciHV-1 cases in white sturgeon were reported for America and Europe (Italy) [28,29]. These insights are reported in phylogenetic studies and did not describe disease symptoms.

Currently, there are no specific tests for detecting AciHV-1. Nevertheless, generic-herpesvirus PCR followed by sequencing are used in phylogenetic studies [28,29,61]. There is no prophylactic or therapeutic treatment for AciHV-1 infection [31].

### 3.2. Acipenserid Herpesvirus 2 (AciHV-2)

Acipenserid herpesvirus 2 (AciHV-2) was isolated few years after sturgeon herpesvirus AciHV-1 was detected. AciHV-2 is currently the only sturgeon herpesvirus officially named by the ICTV and added to the *Ictalurivirus* genus (family *Alloherpesviridae*, order *Herpesvirales*) [31]. AciHV-2 was firsts reported in 1991 from white sturgeon (*A. transmontanus*) ovarian fluid: the fish were farmed in California and showed no clinical signs [30]. The virus was visualized by testing the sample on a cell monolayer derived from a white sturgeon spleen cell line (WSS-2). An experimental trial described in the same study showed a higher mortality rate (up to 80%) compared to AciHV-1 (35%), suggesting greater AciHV-2 virulence [30]. Subsequent geographical expansion (Idaho, Oregon and Wisconsin in the United States and northeastern Canada) and host spectrum expansion (*A. brevirostrum*, *A. fulvescens*) were reported [18,28,29,31]. AciHV-2 susceptibility has been experimentally demonstrated in pallid sturgeon (*S. albus*) and shovelnose sturgeon (*S. platorynchus*) [32]. AciHV-2 has also been reported for Europe: a massive mortality outbreak involving Siberian sturgeon (*A. baerii*) juveniles occurred in Russia. The same causative agent, called Siberian sturgeon herpesvirus (SbSHV), also affected Bester juveniles (*A. ruthenus* × *H. huso*) and Siberian sturgeon adults [33]. Experimental infections in Siberian sturgeon resulted in the total death of juveniles and a high cumulative mortality (36%) also in adults [31]. According to the official nomenclature, SbSHV is assumed to be a strain of AciHV2 [62,63], which is why it is included in this section.

AciHV-2 diagnosis has been carried out on several cell lines (WSSK-1, WSS-2, WSLV, WSGO, SSO-2, SSF-2) that are useful for the isolation and observation of cytopathic effects (focal monolayer destruction, grape-like cell clusters around infective foci) [30,33]. Cell cultures analysis can be coupled with transmission electron microscopy and PCR. There are no molecular specific tests for detecting AciHV-2, although generic herpesviruses PCR may be useful for establishing correct diagnosis [28,29,61,62,64,65]. There are no vaccines or treatment for AciHV-2 [31].

### 3.3. Cyprinid Herpesvirus 3 (CyHV3)

Although not specifically a pathogen of sturgeon, Cyprinid Herpesvirus-3 (CyHV-3) or Koi Herpesvirus (KHV) has been reported in *Acipenseridae*. The two documented cases concern Atlantic sturgeon (*A*. *oxyrhynchus*), Russian sturgeon (*A. gueldenstaedtii*) [34] and a Bester hybrid (*A. ruthenus* x *H. huso*) [35]. Since only viral DNA was found, there is not enough evidence to support the infection hypothesis, as indicated by the World Organization for Animal Health (OIE) manual (“species with incomplete evidence for susceptibility”) [66]. These reports have relevance, however because sturgeon may be a potential reservoir of this notifiable disease.

## 4. White Sturgeon Adenovirus 1 (WSAdV-1)

Although adenoviruses don’t seem to be a problem for sturgeon farming, they have been identified only in sturgeon. Adeno-like particles have, in fact, been seen with electron microscopy in different species (*Gadus morhua*, *Limanda limanda*, *Pagrus major*, *Anguilla japonica*) [67,68,69,70], but White Sturgeon Adenovirus 1 (WSAdV-1) is the only adenovirus classified by the ICTV in fish [71].

The first report dates from 1984 and describes adeno-like particles observed in 0.5 g farmed white sturgeon (*A. transmontanus*) juveniles from California [36]. The study only reported microscopic observation and symptoms since virus isolation on several cell lines (BF-2, BB, FHM, CCO, CHSE-214, SH-1, SS-2) gave negative results. A second case was reported by La Patra and co-authors [37], again in white sturgeon, in which case the virus was isolated on cell monolayer and infected wild sturgeon (Columbia River). The first molecular study of this virus showed a difference between sturgeon isolates and isolates from cold-blooded animals and provided a PCR method targeting a fragment of the adenoviral DNA polymerase gene (GenBank accession number AY082701) [72]. Subsequent phylogenetic studies confirmed this observation [73], leading to the creation of a new genus named *Ichtadenovirus*, of which WSAdV-1 is the type species [71]. Recently, the WSAdV-1 genome has been entirely sequenced (GenBank accession number MK101347) [74]. Experimental infection was reported [36], but no information on immunity is available [75].

For diagnostic purposes, the main symptoms are lethargy, anorexia and emaciation, with a cumulative mortality of 50%. Autopsy reveals pale liver and empty intestines. Virions can be detected by electron microscopy in the nuclei of the spiral valve and straight intestine. Symptoms were observed only in white sturgeon juveniles [36,37]. WSAdV-1 grows on a white sturgeon splenic epithelial cell line (WSS-2) [72]. A PCR protocol is available [72], although there are no reports indicating its use for diagnostic purposes.

## 5. Other Viral Diseases

Infectious hematopoietic necrosis (IHN) is caused by a *Novirhabdovirus* which typically infects salmonids. It is a notifiable disease [76]. A study showed that white sturgeon (*A. transmontanus*) was susceptible to IHN infection producing neutralizing antibodies [38]. The OIE defines sturgeon as a “species with incomplete evidence for susceptibility” [76]. Nonetheless, attention and controls are mandatory, because sturgeon are bred in facilities that can contain IHN-susceptible species (e.g., rainbow trout) [41].

Another disease is spring viremia of carp (SVC), caused by a virus of the *Rhabdoviridae* family. Vicenova and co-authors reported a case of SVCV in Siberian sturgeon (*A. baerii*) from a Czech farm [39]. The sturgeons were reared concomitantly with cyprinids and presented symptoms and mortality. Diagnosis was made by virus isolation on cell monolayer (EPC, FHM, RTG) and identification with ELISA and PCR.

Among the riboviruses, betanodavirus is the causative agent of viral nervous necrosis (VNN) or viral encephaloretinopathy. The virus is a known fish pathogen, particularly in marine species [77]. A case of betanodavirosis in Russian sturgeon (*A. gueldenstaedtii*) was reported in Greece [40]. The sturgeon weighted about 500 g and showed neurological signs; betanodavirus was detected by PCR in brain samples. Compared to the other fish reported in the same work (e.g., sea bass), no virus was found in the eyes of the sturgeon.

Finally, infectious pancreatic necrosis (IPN) in Siberian sturgeon (*A. baerii*), papova-like virus infection in white sturgeon (*A. transmontanus*) and a reovirus of the *Aquareoviridae* family in Chinese sturgeon (*A. sinensis*) have been reported [32,41], but no other data are available. Reovirus in Chinese sturgeon represents the only currently available report of virosis in *Acipenseridae* reported in China.

## 6. Conclusions

The increase in intensive sturgeon farming has led to a consequent increase in infectious diseases. Viral infections are the leading cause of economic loss [1], although there are no statistics quantifying the amount. The main viruses affecting sturgeon are sNCLDV and Alloherpesviruses. Other specific (e.g., Adenoviruses) and non-sturgeon specific viral agents have been found in *Acipenseridae*. The diseases caused by these viruses mainly affect juveniles and can lead to high cumulative mortality [21,26,30,33]. Moreover, cases of viral infection in larger sturgeon are documented.

Currently available information on sturgeon virosis is scarce; the criticalities are:

- Lack of recognition of specific clinical signs of disease;
- Failure to isolate most viral agents;
- Fragmentary genomic data;
- Lack of specific tests and biomolecular methods to identify the causative agent.

These criticalities are interconnected: the lack of recognition of specific clinical signs leads to underestimation of disease, which is further complicated by the inapparent latent infection of certain viruses (e.g., AciHV-1, AciHV-2) [31]. In suspected viral disease, isolation of many etiological agents is often impossible, especially of sNCLDV [21,22,42]. This inevitably precludes conducting extensive virus studies and reflects the lack of data on sturgeon immune response and the real pathological meaning of viral infection. For example, a phylogenetic investigation showed that sNCLDV divides into different clusters in relation to the outbreak area [44]. Although this information adds to our epidemiological knowledge, the pathological role of several viruses remains to be clarified, as does the modality of transmission. Although it has been hypothesized for various sturgeon viruses, vertical transmission of an etiological agent has not been yet demonstrated. Moreover, good animal husbandry practices and health monitoring before and after the importation of new sturgeon batches are necessary for viral disease control. The voluntary nature of health monitoring of viral disease leads to underestimation of the prevalence of infection. In this context, the total absence of reports from China, which is currently the main global producer of sturgeon, is surprising [7].

In conclusion, to obtain information on viral diseases in sturgeon, the first step is the diagnostic process implementation. The development of sturgeon cell lines may be useful for the isolation of many viruses as possible. If isolation is not possible, specific PCRs are the most efficient tool for establishing a rapid diagnosis. To achieve this, close collaboration between breeders and researchers is needed. The study of sturgeon viruses must find ways of preventing and treating these diseases, in order to reduce farming losses and not affect stocking programs.

## Figures and Tables

**Table 1:** Viruses found in various sturgeon species, with relative bibliographical reference.

Virus *		Sturgeon Species	Reference
sNCLDV	WSIV	*A. transmontanus*	[11,12,13,14]
		*A. fulvescens*	[15]
		*A. ruthenus*	[16]
		*A. baerii*	
		*A. gueldenstaedtii*	
		*A. oxyhrynchus*	
		*A. sturio*	
	MRSIV	*S. albus*	[17]
		*S. platorynchus*	
	SNSV	*A. brevirostrum*	[18]
	BCWSV	*A. transmontanus*	[19]
	NV	*A. fulvescens*	[20]
	AcIV-E	*A. gueldenstaedtii*	[21,22,23]
		*A. baerii*	[21,22,24]
		*A. naccarii*	[21]
		*H. huso*	
		*A. stellatus*	[25]
		*A. ruthenus*	
	FV3	*S. albus*	[26]
*Alloherpesviridae*	AciHV-1	*A. transmontanus*	[27,28,29]
	AciHV-2	*A. transmontanus*	[28,29,30]
		*A. brevirostrum*	[18]
		*A. fulvescens*	[31]
		*S. albus*	[32]
		*S. platorynchus*	
		*A. baerii*	[33]
		Bester *(A. ruthenus* x *H. huso)*	
	CyHV-3	*A. gueldenstaedtii*	[34]
		*A. oxyhrynchus*	
		Bester (*A. ruthenus* x *H. huso)*	[35]
*Adenoviridae*	WSAdV-1	*A. transmontanus*	[36,37]
*Rhabdoviridae*	IHNV	*A. transmontanus*	[38]
	SVCV	*A. baerii*	[39]
*Nodaviridae*	Betanodavirus	*A. gueldenstaedtii*	[40]
*Birnaviridae*	IPNV	*A. baerii*	[32]
*Reoviridae*	Aquareovirus	*A. sinensis*	[32]
	Papova-like virus	*A. transmontanus*	[41]

* WSIV: white sturgeon iridovirus; MRSIV: Missouri River sturgeon iridovirus; SNSV: shortnose sturgeon virus; BCWSV: British Columbia white sturgeon virus; NV: namao virus; AcIV-E: acipenser iridovirus-European; FV3: frog virus 3; AciHV-1: acipenserid herpesvirus 1; AciHV-2: acipenserid herpesvirus 2; CyHV-3: cyprinid herpesvirus-3; WSAdV-1: white sturgeon adenovirus 1; IHNV: infectious hematopoietic necrosis virus; SVCV: spring viraemia of carp virus; IPNV: infectious pancreatic necrosis virus.
